# GnRH pulse generator frequency is modulated by kisspeptin and GABA‐glutamate interactions in the posterodorsal medial amygdala in female mice

**DOI:** 10.1111/jne.13207

**Published:** 2022-10-28

**Authors:** Geffen Lass, Xiao Feng Li, Margaritis Voliotis, Ellen Wall, Ross A. de Burgh, Deyana Ivanova, Caitlin McIntyre, Xian‐Hua Lin, William H. Colledge, Stafford L. Lightman, Krasimira Tsaneva‐Atanasova, Kevin T. O'Byrne

**Affiliations:** ^1^ Department of Women and Children's Health, Faculty of Life Sciences and Medicine King's College London London UK; ^2^ Department of Mathematics and Living Systems Institute, College of Engineering, Mathematics and Physical Sciences University of Exeter Exeter UK; ^3^ Reproductive Physiology Group, Department of Physiology, Development and Neuroscience University of Cambridge Cambridge UK; ^4^ The International Peace Maternity and Child Health Hospital, School of Medicine Shanghai Jiao Tong University Shanghai China; ^5^ Henry Wellcome Laboratories for Integrative Neuroscience and Endocrinology, The Dorothy Hodgkin Building University of Bristol Bristol UK

**Keywords:** amygdala, GABA, glutamate, kisspeptin, LH

## Abstract

Kisspeptin neurons in the arcuate nucleus of the hypothalamus generate gonadotrophin‐releasing hormone (GnRH) pulses, and act as critical initiators of functional gonadotrophin secretion and reproductive competency. However, kisspeptin in other brain regions, most notably the posterodorsal subnucleus of the medial amygdala (MePD), plays a significant modulatory role over the hypothalamic kisspeptin population; our recent studies using optogenetics have shown that low‐frequency light stimulation of MePD kisspeptin results in increased luteinsing hormone pulse frequency. Nonetheless, the neurochemical pathways that underpin this regulatory function remain unknown. To study this, we have utilised an optofluid technology, precisely combining optogenetic stimulation with intra‐nuclear pharmacological receptor antagonism, to investigate the neurotransmission involved in this circuitry. We have shown experimentally and verified using a mathematical model that functional neurotransmission of both GABA and glutamate is a requirement for effective modulation of the GnRH pulse generator by amygdala kisspeptin neurons.

## Graphical abstract

Modelling amygdala upstream regulation of the hypothalamic GnRH pulse generator (ARC KNDy network). (A), Optogenetic stimulation (a) of MePD Kiss1 neurones activates the GABA‐GABA disinhibitory pathway (b) reducing the inhibitory GABAergic tone arising from the MePD (c), as well as activating glutamatergic interneurones (d) projecting to the GABAergic efferents, counteracting the stimulatory output of the MePD (e) to accelerate GnRH pulse generator frequency. (B), GABA receptor antagonism in the MePD with optical stimulation of MePD Kiss1, results in net inhibition of the KNDy network to decrease pulse frequency. (C), Glutamate receptor antagonism with optical stimulation of MePD Kiss1, over‐stimulates the KNDy network resulting in a transition from a pulsatile to a quiescent dynamic state of the GnRH pulse generator.

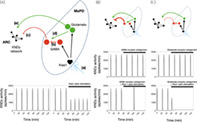

## 1 INTRODUCTION

Recent investigations have revealed a significant stimulatory role for kisspeptin in the posterodorsal subnucleus of the medial amygdala (MePD) in gonadotrophin‐releasing hormone (GnRH) pulse generator modulation. Our previous optogenetic studies showed that sustained low‐frequency blue light stimulation activated these neurons to increase luteinising (LH) pulse frequency, a proxy for GnRH pulse generator frequency.^1^ This finding built upon our previous neuropharmacological approach using intra‐MePD infusions of kisspeptin receptor (Kiss1r) agonists and antagonists, which respectively increased LH secretion or decreased LH pulse frequency.^2^ However, the mechanisms underlying this neuronal population's stimulatory role over pulsatile LH secretion have not been studied. Glutamate and GABA are the major stimulatory and inhibitory neurotransmitters in the mammalian brain, and many neuronal networks rely on the balance between these two to regulate their activity.^3^ Therefore, these two neurotransmitters are sensible candidates to be used by the amygdala neuronal networks underlying the upstream, extra‐hypothalamic regulation of the GnRH pulse generator. Unsurprisingly, both GABA and glutamate neurons are found in the MePD,^4^, ^5^ and pharmacological antagonism of both has deleterious effects on several aspects of reproductive physiology.^6^, ^7^, ^8^ In rats, blocking AMPA and NMDA glutamate receptors with CNQX and AP5, respectively, impedes activation of MePD neurons in response to vaginal‐cervical stimulation, thereby preventing the pregnancy or pseudopregnancy response following intromission; AMPA antagonism also disrupts oestrous cycles.^6^, ^7^ Furthermore, MePD NMDA and GABA_A_ receptor (GABA_A_R) antagonism causes a weight‐independent advancement of puberty.^8^ A reciprocal relationship between kisspeptin and GABA in the limbic system has also been shown, with i.v. kisspeptin administration in humans resulting in a reduced GABA signal in the anterior cingulate cortex.^9^ Thus, GABA and glutamate play an important role in the MePD in reproductive physiology.

The interesting dichotomy between the well‐known suppressive role of the MePD in reproductive physiology and the emerging activatory function of kisspeptin within this amygdaloid subnucleus has led to a hypothesis that MePD kisspeptinergic activity may stimulate GABAergic interneurons within the MePD that in turn synapse with, and inhibit, GABAergic projection efferents from the MePD, resulting in an overall disinhibition of the latter. Evidence to support this hypothesis stems from the knowledge that there is a significant population of GABAergic neurons that project from the MePD to reproductive neural centres such as those in the hypothalamus,^4^, ^10^ and inhibitory GABA interneurons, specifically, have also been detected in this subregion.^11^, ^12^ This is in line with the fact that the MePD is a pallidal subnucleus, as a result of its embryological origins in the caudoventral medial ganglionic eminence, indicating its similarity to other neural complexes which contain a classical GABA‐GABA disinhibitory system.^13^ Furthermore, other subnuclei of the amygdala, such as the basolateral amygdala and posteroventral medial amygdala, have been shown to exhibit functional glutamatergic signalling onto GABA interneurons,^12^, ^14^, ^15^ supporting the hypothesis of an alternative glutamate‐GABA‐GABA pathway by which kisspeptin may activate the disinhibitory system.

It is therefore critical to investigate the GABAergic and glutamatergic signalling within the MePD with respect to kisspeptin and its action over GnRH pulse generator activity. To achieve this, a dual approach of simultaneously combining optogenetic activation and pharmacological antagonists was used via the implantation of an intra‐MePD optofluid cannula. By optically stimulating the kisspeptin neurons in the presence or absence of glutamate or GABA antagonists during frequent blood sampling for measurement of LH pulses, it could be determined whether either of these neurotransmitters are involved in the GnRH pulse generator modulating role of MePD kisspeptin. Additionally, we used mathematical modelling to interrogate potential interactions between GABAergic and glutamatergic neurons within the MePD and their projections to the arcuate nucleus to modulate GnRH pulse generator frequency.

## 2 MATERIALS AND METHODS

### 2.1 Animals

Breeding pairs of Kiss‐Cre^+/−^:tdTomato^+/+^ transgenic mice were obtained from the Department of Physiology Development and Neuroscience, University of Cambridge, Cambridge, UK; the Kiss‐CRE mice carry a tdTomato transgene activated by CRE to label MePD Kiss1 neurons. Litters from breeding pairs were genotyped using a multiplex polymerase chain reaction (PCR) protocol to detect heterozygosity for the Kiss‐Cre or wild‐type allele as described previously.^1^, ^16^ Adult female mice (19–23 g), heterozygous for the Kiss‐Cre transgene were individually housed under controlled conditions (12:12 h light/dark photo cycle, lights on 07:00 am, 25 ± 1°C) with access to food and water available ad libitum. All procedures were approved by the Animal Welfare and Ethical Review Body (AWERB) Committee at King's College London, in accordance with the United Kingdom Home Office Animals (Scientific Procedures) Act 1986.

### 2.2 Stereotaxic injection of channelrhodopsin viral construct and implantation optofluid cannula

All surgical procedures were carried out under a combination of ketamine anaesthesia (Ketamidor, 100 mg kg^−1^, i.p.; Chanelle Vet, Hungerford, UK) and xylazine (Rompun, 10 mg kg^−1^, i.p.; Bayer, Leverkusen, Germany) under aseptic conditions. Mice were bilaterally ovariectomised (OVX) to mitigate negative feedback of endogenous oestrogen on LH secretion. Stereotaxic injection of the viral construct and implantation of the brain cannula was carried out concurrently with ovariectomy. Mice were placed in a Kopf motorised stereotaxic frame (Kopf, Tujinga, CA, USA) and procedures were carried out using a robot stereotaxic system (Neurostar, Tubingen, Germany). Following an incision of the scalp, a small hole was drilled into the skull at a location above the MePD. The stereotaxic injection coordinates used to target the MePD were obtained from the mouse brain atlas of Paxinos and Franklin^17^ (2.1 mm lateral, 1.70 mm posterior to bregma and at a depth of 5.1 mm). Using a 2‐μL Hamilton micro‐syringe (Esslab, Westcliff‐on‐Sea, UK) attached to the stereotaxic frame micro‐manipulator, 0.4 μl of the ChR2 virus, AAV9‐EF1a‐double floxed‐hChR2(H134R)‐EYFP‐WPRE‐HGHpA (≥ 1 × 10^13^ vg mL^−1^; Addgene, Watertown, MA, USA) was injected unilaterally into the right MePD over 10 min. The needle was left in position for a further 5 min and then slowly withdrawn over 1 min. Following the injection of adeno‐associated virus (AAV), mice heterozygous for Kiss‐Cre were implanted with a dual optofluid cannula (Doric Lenses, Québec, QC, Canada) at the same anteroposterior and mediolateral coordinates as the virus injection site, but at dorsoventral coordinates such that the internal cannula targets the MePD and the fibre optic cannula is situated 0.25 mm above. Dental cement (Superbond C&B kit; Prestige Dental Products, Bradford, UK) was used to fix the cannula in place and the skin incision was closed with a suture. Antibiotics were administered prophylactically post‐surgery (Betamox, 0.15 mg g^−1^, s.c; Norbrook Laboratories, Newry, UK). Mice were left for 4 weeks preceding the experimental period to allow for effective opsin expression in target regions.

### 2.3 Blood sampling procedure for LH measurement

Following a 1‐week recovery period from surgery, the mice were handled daily to acclimatise them to the tail‐tip blood sampling procedure for measurement of LH.^1^ The blood samples were processed via an enzyme‐linked immunosorbent assay as reported previously.^18^ Mouse LH standard (AFP‐5306A; dilution 1:25) and primary antibody (AFP240580Rb; dilution 1:40) were purchased from Harbour‐UCLA (West Carson, CA, USA) and secondary antibody (NA934; no dilution) was from VWR International (Lutterworth, UK). The intra‐assay and inter‐assay variations were 4.6% and 10.2%, respectively.

### 2.4 In vivo optogenetic stimulation of MePD kisspeptin neurons and intra‐MePD infusion of bicuculline, CGP‐35348 or CNQX + AP5

On the day of the experiment, the zirconia ferrule of the implanted cannula was attached via a ceramic mating sleeve to a multimode fibre optic rotary joint patch cable (Thorlabs Ltd, Ely, UK) at a length that allowed for free movement of the mice in their home cage. Blue light (473 nm wavelength; 5 mW) was delivered using a Grass SD9B stimulator‐controlled laser (Laserglow Technologies, Toronto, ON, Canada). Following 1 h of acclimatisation, blood samples (4 μL) were collected every 5 min for 2.5 h. The first hour of blood collection consisted of no stimulation and, in the subsequent 1.5 h, Kiss‐Cre mice received optic stimulation at 5 Hz; this stimulation protocol was chosen following previous work showing this frequency to be the most efficacious in increasing LH pulsatility in OVX mice.^1^

For the neuropharmacological manipulation of GABA or glutamate receptor signalling with or without simultaneous optogenetic stimulation, mice were connected to the laser as described above but, additionally, an injection cannula connected to extension tubing preloaded with drug solution was inserted into the guide cannula of the optofluid implant immediately after connection of the fibre optic cannula. At 10 min before optic stimulation, bolus administration of selective GABA_A_R (bicuculline; Sigma‐Aldrich, St Louis, MO, USA), GABA_B_R (CGP‐35348; Sigma‐Aldrich) or a cocktail of both NMDAR and AMPAR glutamate receptor (AP5; Tocris, Bristol, UK; CNQX; Tocris) antagonist dissolved in artificial cerebrospinal fluid (aCSF) commenced and continued over 5 min. The concentrations for the bicuculline, CGP‐35348, AP5, and CNQX boli were 20 pmol, 4.5 nmol, 1.2 nmol and 0.5 nmol respectively. After 10 min, immediately prior to the onset of optogenetic stimulation, continuous drug infusion commenced and continued for the remainder of the experiment (1.5 h). The total concentrations for the continuous infusion of bicuculline, CGP‐35348, AP5 and CNQX were 68 pmol, 15 nmol, 2 nmol and 1 nmol respectively. The same regimen was used in the absence of optic stimulation. As a control, Kiss‐Cre mice received aCSF (0.3 μL bolus, 1 μL continuous) in the presence of optic stimulation at 5 Hz following the same timeframe as the test experiments. The concentrations of antagonists used were based on our previous research and our preliminary studies.

### 2.5 Validation of AAV injection site

Upon completion of experimental procedures, the mice were killed with a lethal dose of ketamine and transcardially perfused with heparinised saline for 5 min, followed by 10 min of ice‐cold 4% paraformaldehyde in phosphate‐buffered saline (pH 7.4) for 15 phosphate‐buffered saline using a pump (Minipuls, Gilson, Villiers Le Bel, France). Their brains were rapidly collected and post‐fixed sequentially at 4°C in 15% sucrose in 4% paraformaldehyde and in 30% sucrose in 1 × phosphate‐buffered saline until they sank. Brains were then snap frozen on dry ice and stored at −80°C until processing. Coronal brain slices (30 μm thick) were sectioned using a cryostat (Bright Instrument, Luton, UK). Every third section was collected between −1.34 mm and −2.79 mm from the bregma. Sections were mounted on glass microscope slides, air‐dried and cover‐slipped with Prolong Antifade mounting medium (Molecular Probes, Inc., Eugene, OR, USA). Only animals expressing enhanced yellow fluorescent protein (EYFP) in the MePD were analysed by using Axioskop 2 Plus microscope equipped with Axiovision, version 4.7 (Zeiss, Oberkochen, Germany).

### 2.6 Statistical analysis

Appropriate sample sizes were predetermined by way of power analyses that were performed using the SigmaStat software (Systat Software Inc., San Jose, CA, USA) and expected variances and effect sizes that were based on our preliminary and published studies.^1^ The Dynpeak algorithm was used to establish LH pulses.^19^ The effect of optogenetic stimulation and neuropharmacology studies was established by comparing the mean LH interpulse interval (IPI), from the 1 h pre stimulation or drug administration control period to the 1.5 h experimental period. IPI are reported as change from the baseline condition for each mouse. On occasions where there were no LH pulses observed in the post treatment interval, the IPI was given a value of 90 min. LH pulse parameters were analysed by a two‐way repeated measures analysis of variance (time and AAV condition and/or drug administration, respectively) and a subsequent Tukey's post‐hoc test. All statistics were performed using SigmaPlot, version 14 (Systat Software Inc.). Data are presented as the mean ± SEM. *p* < .05 was considered statistically significant.

### 2.7 Mathematical model

A previously published mathematical model of the arcuate nucleus (ARC) Kiss1 population^20^, ^21^ was extended to incorporate MePD regulation. This extended model was used to test our hypothesis regarding interactions within the MePD circuitry. The extended model incorporates: (i) the disinhibitory GABA‐GABA pathway from the MePD; (ii) glutamatergic interneurons that in turn project to the GABA‐GABA pathway; and (iii) glutamatergic projections from the MePD onto the ARC. The model was implemented and run in MATLAB. The model along with the parameter values used for the simulations presented can be downloaded from https://git.exeter.ac.uk/mv286/extended-kndy-model.

## 3 RESULTS

### 3.1 Validation of AAV injection site and cannula position

**FIGURE 1:**
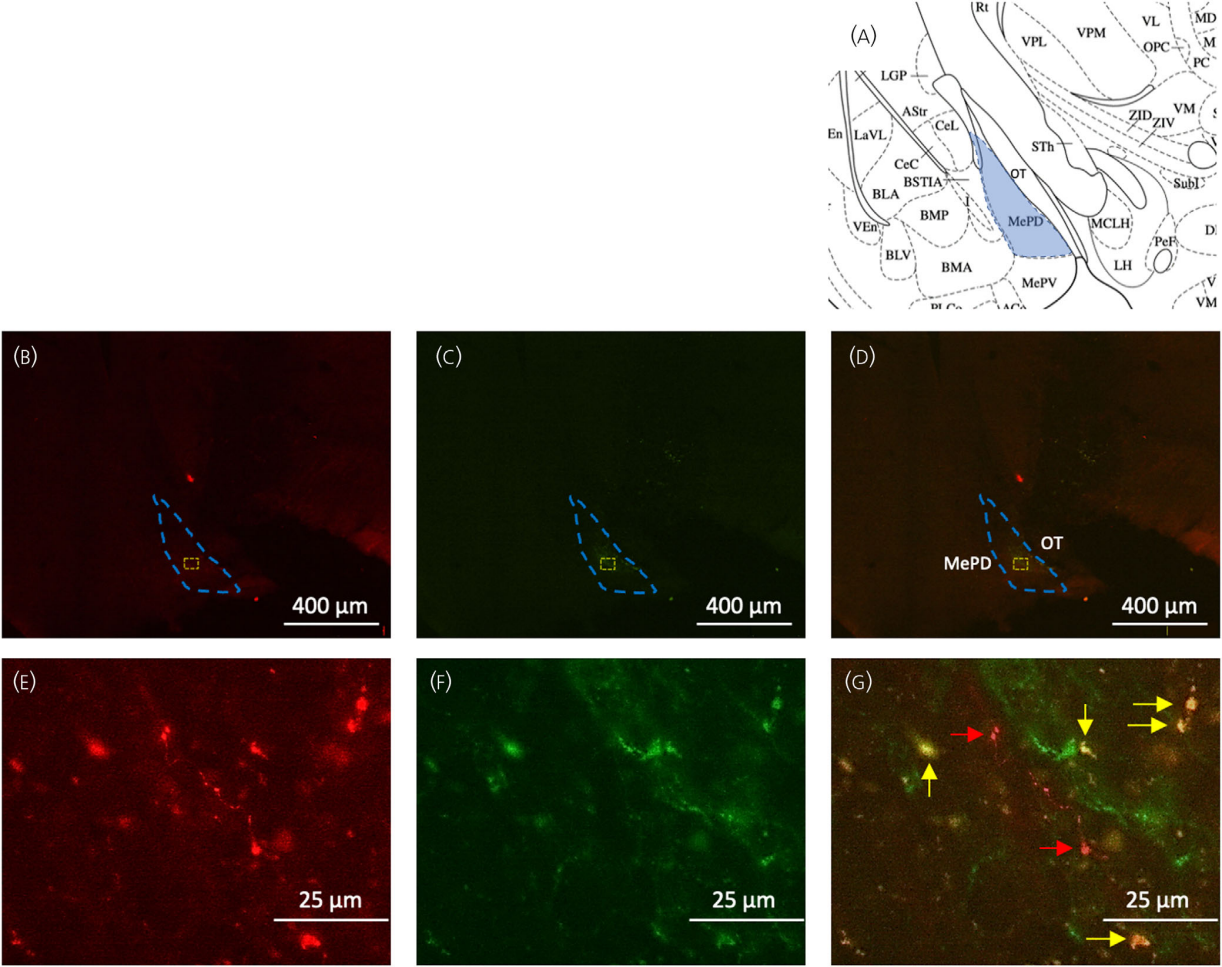
Expression of ChR2‐EYFP in medial amygdala (MePD) kisspeptin neurones in Kiss‐Cre mice. (A) Aschematic representation of the MePD and its spatial relationship with the optic tract (OT). (B) tdTomato‐expressing kisspeptin neurones are shown, whereas (C) shows those cells infected with enhanced yellow fluorescent protein (EYFP). Coronal section showing green EYFP fluorescence positive neurones in the MePD (D). (E–G) Hhigher‐power magnification of the area in (B–D) encased with the yellow dotted line, showing fluorescence of tdTomato (red cells), EYFP (green cells) and both (yellow cells), respectively. MePD kisspeptin neurones tagged with EYFP (labelled with yellow arrows) and not tagged with EYFP (red arrows) are shown in (G). OT, oxytocin

The AAV‐ChR2 virus used to infect the cells in Kiss‐Cre mice was tagged with fluorescent EYFP to be visualised under a microscope. The mean ± SEM number of tdTomato‐expressing kisspeptin cells in unilaterally‐injected brain sections was 24.50 ± 5.20 per animal and 20.33 ± 4.50 (~83%) of tdTomato‐expressing neurons were EYFP positive. Analysis of images acquired from coronal sectioning of the mouse brains showed that seven out of nine animals had successful stereotaxic injection of AAV‐ChR2 virus into the MePD, and all seven also had successful cannula implantation into the MePD. A representative example of a coronal brain section is shown in Figure 1. Dual fluorescence labelling revealed EYFP infection in tdTomato (Kiss‐Cre‐expressing neurons) cell bodies (Figure 1G). Of note there is some non‐specific expression of ChR2 in the MePD (YFP single labelling). The LoxP sites in FLEX plasmid vectors are known to recombine during DNA amplification and viral vector production, which may result in a minority of Cre‐activated (i.e., “flipped”) viral vectors (Vector number 20298; Addgene). Despite our attempts to reduce the likelihood of Cre‐independent expression by optimising the injection volume and viral titres, we cannot exclude the fact that non‐specific expression has occurred although this is minimal compared to the number of MePD Kiss1 neurons infected. In addition, it is likely some of the YFP staining not colocalised with tdTomato is debris as a result of the time‐length of our in vivo experiment.

### 3.2 Effects of sustained optical stimulation at 5 Hz, with and without a control administration of aCSF, on LH pulse frequency

**FIGURE 2:**
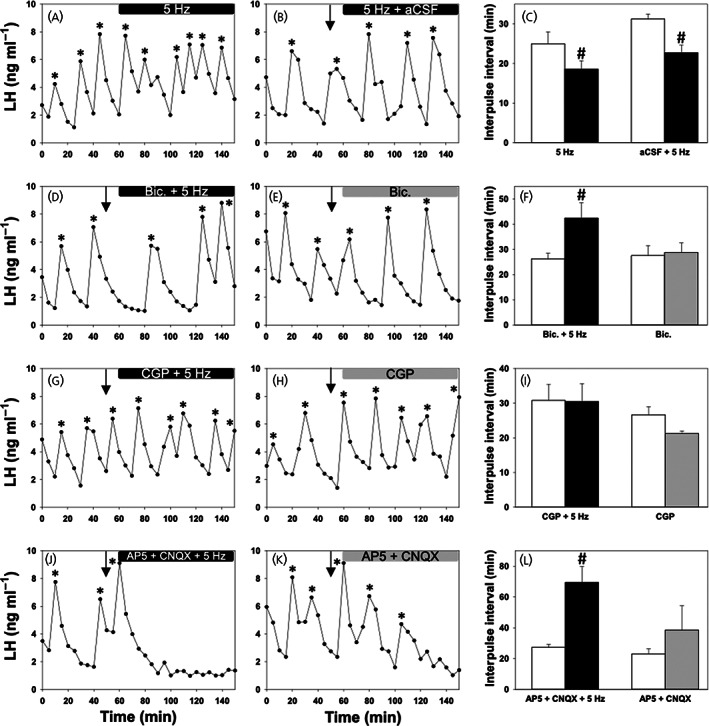
Effect of GABA and glutamate receptor antagonism with and without sustained optogenetic stimulation of medial amygdala (MePD) Kiss1 neurones on luteinising hormone (LH) pulse frequency. Representative examples showing the effects of stimulation at 5 Hz (A), 5 Hz + artificial cerebrospinal fluid (aCSF) (B), bicuculline +5 Hz (D), CGP‐35348 + 5 Hz (G) and AP5 + CNQX +5 Hz (J) on pulsatile LH secretion in ovariectomised mice. Representative examples for the effects of drugs alone – bicuculline (E), CGP (H) and AP5 + CNQX (K) – are also shown. Histograms showing the mean LH interpulse interval for all interventions are also provided (C, F, I, L). Sustained stimulation at 5 Hz in the presence (*n* = 4) and absence (*n* = 7) of aCSF infusion resulted in a significant reduction in the LH interpulse interval (*p* < 0.05). Sustained stimulation together with bicuculline (*n* = 5) and AP5 + CNQX (*n* = 6) resulted in a significant increase in LH interpulse interval. Wild‐type control animals did not respond to stimulation. LH pulses detected by the DynPeak algorithm are indicated with an asterisk. Bolus injections of antagonists administered over 5 min are indicated by downward black arrows at the 50‐min time point and were followed by continuous drug infusions at the 60‐min time point indicated by the horizontal black (optical stimulation + antagonist or aCSF) or grey (antagonist alone) bars. *p < .05 versus, control. Data represent the mean ± SEM

After a 1‐h control blood sampling period in the absence of optical stimulation, Kiss‐Cre mice were stimulated at 5 Hz for 90 min with and without administration of aCSF. In both experimental protocols, the stimulation resulted in a significant increase in LH pulse frequency (Figure 2A, B). The mean IPI decreased from 25.00 ± 2.99 min to 18.60 ± 2.07 min (AAV‐ChR2 stimulation significant interaction; *F*_1,21_ = 6.04, *n* = 7; *p* = .002) after stimulation at 5 Hz only, and from 31.25 ± 1.25 min to 22.73 ± 1.94 min (AAV‐ChR2 stimulation significant interaction; *F*_1,21_ = 8.45, *n* = 4; *p* = .001) after stimulation at 5 Hz and infusion of aCSF (Figure 2C). However, there were no statistic significant differences in the means between these two groups at the same time point (interaction between groups was not significant; *F*_1,21_ = 0.72, *p* = .418).

### 3.3 Effects of bicuculline, a GABA_A_R antagonist, on LH pulse frequency in the presence and absence of sustained 5‐Hz optical stimulation

In the second part of the experiment, Kiss‐Cre mice received a unilateral intra‐MePD infusion of bicuculline with and without sustained 5‐Hz optical stimulation. After the dual treatment of light and bicuculline, the average LH IPI significantly increased from 27.00 ± 2.55 min to 42.42 ± 7.39 min (significant interaction; *F*_1,21_ = 7.27, *n* = 5; *p* = .008), indicating reduced LH pulse frequency (Figure 2D, F). There was no significant change in the IPIs before and after bicuculline administration alone, with a pre‐infusion IPI of 29.00 ± 3.78 min and a post‐infusion IPI of 29.75 ± 3.78 min (interaction was not significant; *F*_1,21_ = 1.284, *n* = 5; *p* = .771) (Figure 2E, F).

### 3.4 Effects of CGP, a GABA_B_R antagonist, on LH pulse frequency in the presence and absence of sustained 5‐Hz optic stimulation

In the third part of the experiment, Kiss‐Cre mice received a unilateral infusion of CGP‐35348, an antagonist selective for the GABA_B_R, both with and without continuous optogenetic stimulation. There was no significant difference in LH IPI between the initial 60‐min control period and subsequent period of intervention in either of these experimental protocols; however a trend towards increased LH pulse frequency during administration of CGP‐35348 alone in the absence of light was observed (Figure 2G–I). The pre‐ and post‐intervention average IPIs for CGP‐35348 with stimulation at 5 Hz were 30.80 ± 4.67 min and 30.53 ± 5.10 min, respectively (no significant interaction; *F*_1,15_ = 1.22, *n* = 5; *p* = .954). For CGP‐35348 administration alone, the pre‐ and post‐intervention average IPIs were 26.67 ± 2.28 min and 21.25 ± 0.72 min, respectively (no significant interaction; *F*_1,15_ = 2.23, *n* = 5; *p* = .115).

### 3.5 Effects of glutamate receptor antagonism on LH pulse frequency with and without continuous 5‐Hz optic stimulation

The final protocol of the experiment involved unilateral infusion of a drug cocktail consisting of both AP5 and CNQX, antagonists for AMPA and NMDA receptors, respectively, in the presence and absence of light in Kiss‐Cre mice (Figure 2J–L). After the 60‐min control period, sustained 5‐Hz optogenetic stimulation together with infusion of the antagonist resulted in a significantly decreased LH pulse frequency; indeed, in a number of cases, LH pulsatility ceased altogether and the average IPI increased from 27.33 ± 1.89 min to 69.50 ± 10.26 min (significant interaction; *F*_1,17_ = 11.90, *n* = 6; *p* = .007). For AP5 and CNQX alone, there was a trend of increased IPI before and after treatment from 23.04 ± 3.24 min to 38.59 ± 15.73 min, although this was not significant (no significant interaction; *F*_1,15_ = 2.53, *n* = 4; *p* = .155).

### 3.6 In silico confirmation of the pulse generator responses to MePD regulation

**FIGURE 3:**
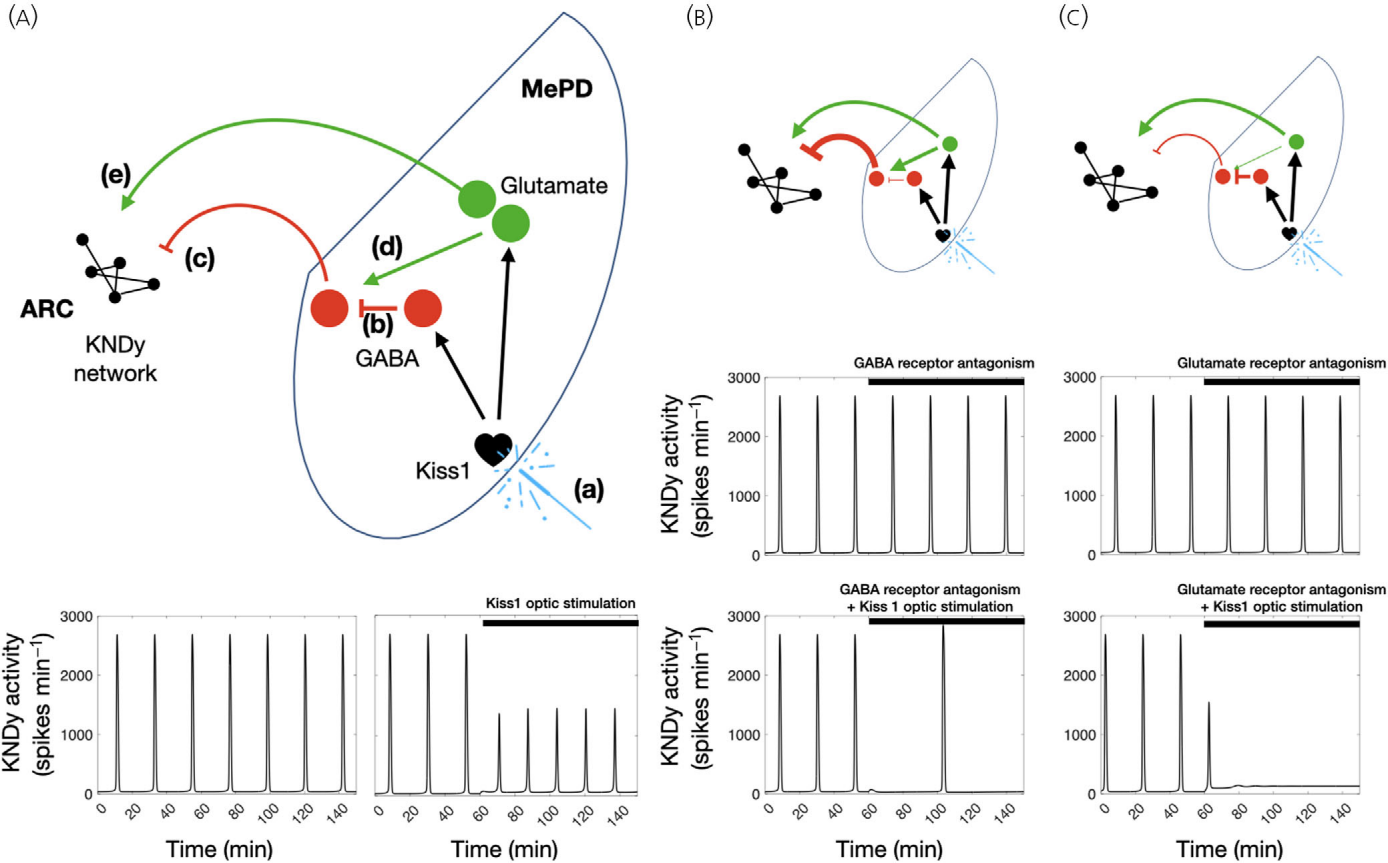
Proposed interactions and pathways involved in the medial amygdala (MePD) regulation over the hypothalamic gonadotrophin‐releasing hormone (GnRH) pulse generator (ARC^KNDy^). (A) According to the hypothesis model, kisspeptin in the MePD regulates the GnRH pulse generator by activating two pathways: (i) a GABA‐GABA disinhibitory pathway and (ii) a pathway involving glutamatergic MePD projection neurones. Optogenetic stimulation of MePD *Kiss1* (a) results in activation of the GABA‐GABA disinhibitory pathway (b) that leads to a reduction in GABAergic tone arising from the MePD (c), as well as amplification of the MePD glutamatergic tone (d); this also causes activation of glutamatergic interneurones that project to the GABAergic efferent neurones, counteracting the stimulatory output of the MePD (e). (B) GABA receptor antagonism in the MePD, combined with the optic stimulation of MePD *Kiss1*, results in net inhibition of the arcuate nucleus (ARC) KNDy (i.e., kisspeptin, neurokinin B and dynorphin) network leading to a decrease in pulse frequency. (C) Glutamate receptor antagonism, combined with the optic stimulation of MePD *Kiss1*, results in over‐stimulation of the ARC KNDy network resulting in a transition from a pulsatile to a quiescent dynamic state of the GnRH pulse generator

To verify the LH pulse frequency response to antagonism of GABA and glutamate receptors in the MePD, we extended our mathematical model of the pulse generator^20^, ^21^ to incorporate MePD inputs. The model of the MePD circuitry that we consider involves glutamatergic and GABA‐GABA disinhibitory projections to the KNDy (i.e., kisspeptin, neurokinin B and dynorphin) network, as well as glutamatergic activation of the GABAergic MePD projections to KNDy neurons (Figure 3A). The model successfully reproduces the increase in LH pulse frequency under sustained activation of MePD Kiss1 neurons (Figure 3A). Furthermore, inhibiting GABAergic activity in MePD during optical stimulation shifts the balance from a stimulatory to a net inhibitory output from the MePD, resulting in reduced pulse frequency (Figure 3B). Finally, antagonism of glutamatergic activity in MePD results in net increase of stimulatory inputs to the KNDy network leading to inhibition of ultradian oscillations, such that the KNDy network undergoes a bifurcation, which terminates pulsatile dynamics, as an upper threshold of input activity is crossed^20^ (Figure 3C). At this point, it must be noted the general agreement that lower (≤ 5 Hz) frequencies of stimulation are less likely to elicit the secretion of neuropeptides from neurone terminals, but more likely to lead to secretion of neurotransmitters such as GABA and glutamate^22^, ^23^, ^24^; therefore, activation of MePD kisspeptin neurones in the present study may be causing the release of neurotransmitters and not kisspeptin per se.

## 4 DISCUSSION

The present study highlights for the first time a potential mechanism by which kisspeptin activity in the MePD is stimulatory over the hypothalamic GnRH pulse generator. Building upon previous findings that low‐frequency (5 Hz) optogenetic stimulation of MePD Kiss1 neurons increases the frequency of pulsatile LH secretion,^1^ the results presented here support the hypothesis that this is reliant upon the activity of both GABA and glutamate in the MePD.

The MePD, with its overwhelmingly GABAergic neuronal outputs, is a significant inhibitor of gonadotrophic hormone secretion and wider facets of reproductive physiology. Stimulation of the MePD can advance pubertal onset, whereas lesioning this region delays puberty.^25^, ^26^ In addition, activation of the MePD during stress is deleterious on reproductive function and behaviour.^27^ However, recent findings indicating that MePD Kiss1 neurons stimulate the GnRH pulse generator raise the hypothesis of intranuclear GABA‐GABA disinhibitory interactions, typical of limbic pallidal structures such as the MePD.^1^ The present study tested this hypothesis by utilising specific optogenetic activation of MePD kisspeptin neurons in conjunction with pharmacological inhibition of GABA receptor signalling using bicuculline and CGP‐35348 (intra‐MePD antagonists for GABA_A_R and GABA_B_R, respectively). Indeed, either of these drugs together with optic stimulation prevented the increase in LH pulse frequency seen with stimulation at 5 Hz alone. The result of intra‐MePD infusion of bicuculline together with 5‐Hz optogenetic stimulation is surprising and poses an interesting question: how can intra‐MePD GABA_A_R antagonism not only prevent the stimulatory effects of optogenetic stimulation, but also cause the opposite result of significantly reducing the activity of the GnRH pulse generator, whereas bicuculline alone has no effect on LH pulse frequency? To start understanding this phenomenon, we extended our mathematical model of the KNDy network^20^, ^21^ to include MePD circuitry involving intranuclear GABA‐GABA disinhibitory interactions and glutamatergic synaptic mechanisms. Indeed, a stimulatory effect of the MePD kisspeptinergic system over the GnRH pulse generator has been linked to glutamatergic activation; the pubertal transition is tightly correlated with a developmental increase in the expression of both kisspeptin^28^ and glutamate^29^ in the MePD, and 29% of MePD Kiss1 neurons in adult male mice co‐express vesicular glutamate transporter 2 mRNA^30^; indeed, the use of glutamate as a neurotransmitter by Kiss1 neurons has been found for the KNDy population^21^ including their regulation of Kiss1 neurons in the AVPV.^24^ The present finding of an almost‐complete ablation of pulsatile LH secretion following MePD kisspeptin activation combined with infusion of intranuclear glutamate antagonists provides further support to MePD kisspeptin effect's dependence on glutamate. Therefore, the following mechanism supported by our mathematical model is proposed: optical stimulation of MePD kisspeptin in the presence of GABA_A_R antagonists decreases LH pulse frequency by glutamatergically activating the hypothetical GABAergic projections from the MePD to KNDy neurons (Figure 3B); whether this occurs via glutamate secretion from MePD kisspeptin cells themselves is unknown. In other words, cancelling the ability of GABA interneurons to take part in the disinhibition during optical stimulation shifts the balance from a stimulatory to inhibitory output from the MePD.

The fact that GABA_A_R antagonism alone failed to cause any change in LH pulse frequency is an important factor in our model: it is possible that under basal conditions MePD kisspeptin neurons are relatively quiescent, and therefore solely pharmacologically blocking the inputs to the GABAergic MePD projections without a corresponding increase in glutamatergic activity would make little change to the net influence of the MePD over the KNDy system. Although silent kisspeptin signalling under basal conditions supports the current hypothesised model, it contradicts neuropharmacology studies that show antagonising endogenous kisspeptin within the MePD causes a robust decrease in LH pulse frequency.^2^ However, this latter study used OVX rats that were supplemented with 17β‐oestradiol to mimic the hormonal profile in the diestrus phase of the oestrous cycle. It is important to note that the present study used OVX mice that were not supplemented with 17β‐oestradiol. Kiss1 expression within the MePD varies in relation to the oestrous cycle with low expression observed in OVX mice; oestradiol treatment, however, amplified Kiss1 expression in this brain region.^31^ This may explain why, under basal conditions, the MePD kisspeptin system appears reduced in our OVX mouse model.

Therefore, the proposed model does well to explain why GABA_A_R antagonism in the presence of MePD Kiss1 optical stimulation reduces LH pulse frequency. However, a possible explanation as to why optogenetically stimulating these neurons in the presence of intra‐MePD glutamate antagonists essentially stops all GnRH pulse generator activity is more complex. Complying with the model would suggest that blocking glutamate activity, at the same time as activating the GABA‐GABA disinhibitory system, would in fact increase LH pulse frequency rather than prohibiting it altogether. Nevertheless, we provide a potential explanation for this phenomenon. By examining all of the individual pulse profiles more closely, a subtle, but potentially crucial aspect is identified. In over 80% (5 out of 6) of tests in which glutamate antagonists were infused in conjunction with optical stimulation, a pulse of LH was detected precisely 10 min after the bolus infusion, and immediately before the onset of light stimulation. Only once the optic laser was switched on did the detection of LH pulses reliably cease. Therefore, it is reasonable to posit that, although this protocol indeed blocked GnRH pulse generator activity, this occurs via a mechanism of potential over‐stimulation which sends the KNDy system into a state of inertia because it is unable to respond. The proposed hypothesis is that activation of MePD kisspeptin drives (i) the disinhibitory GABA‐GABA pathway from the MePD; (ii) glutamatergic interneurons that in turn project to the GABA‐GABA pathway; and (iii) glutamatergic projections from the MePD onto the ARC (Figure 3A). Hence, optogenetic stimulation of the MePD Kiss1 neurons combined with antagonism of MePD glutamate results in the net effect of heightened activation of the GnRH pulse generator and resultant inertia. The proposal of excessive GnRH pulse generator neuronal activity resulting in depolarisation silencing is in line with recent experimental findings: using mathematical models confirmed with in vivo optogenetics, it is now known that the ultradian oscillation of the hypothalamic KNDy network works on a bifurcation system that is eventually terminated as an upper threshold of basal neuronal activity is reached^20^; we have described in detail how stimulation of the KNDy network increases in network excitability (e.g., via glutamatergic activity) or neurokinin B signalling results in a robust transition from a pulsatile to a quiescent dynamic state of the GnRH pulse generator.^21^ Additionally, as the upper bifurcation threshold is approached, the frequency of the pulse generator increases significantly and this could also be contributing to the inhibition of pulsatile LH release via desensitisation at the gonadotroph.^32^ Further work using ovariectomised primates (K. T. O'Byrne, unpublished data) showed that administration of NMDA, a potent neuronal activator, evoked a multiunit electrical activity volley, the electrophysiological correlate of GnRH pulse generator activity^33^ and corresponding LH pulse, followed by neuronal silence and cessation of LH pulses. Thus, the GnRH pulse generator is highly sensitive to incoming stimuli and may be prone to silencing by excessive activation. Importantly, glutamate antagonism alone did not result in a significant decrease in LH pulse frequency, and this is in line with the abovementioned theory of basal quiescence of the MePD kisspeptin system.

The present study also investigated the role of GABA_B_ signalling in the activity of MePD kisspeptin and the GnRH pulse generator using CGP‐35348, a GABA_B_R selective antagonist. By contrast to the significant reduction in LH pulse frequency observed with bicuculline and optic stimulation, the interference of GABA_B_R signalling in conjunction with optogenetics only went so far as to prevent the increase in LH pulsatility, indicating a present, yet smaller, influence. The reason for this difference remains unclear, but can be potentially explained by the pharmacological differences between GABA_A_ and GABA_B_ receptors, with the former accounting for fast inhibition, whereas the latter is responsible for slow inhibition.^34^ Moreover, it has been shown that, in the case of LH release, only i.c.v. activation of the GABA_A_R, and not GABA_B_R, reduced LH release from the pituitary and GnRH levels in the preoptic area,^35^ suggesting a differential role for the two receptor subtypes in reproductive neuroendocrinology. Moreover, although it has been shown that knockout of the GABA_B_R subtype in adult female mice results in subfertile phenotypes such as decreased hypothalamic levels of GnRH and GnRH mRNA, it has no diminishing effects on LH or follicle‐stimulating hormone levels, or Kiss1 expression in the hypothalamus^36^, ^37^, ^38^; Kiss1 levels in the MePD, however, are increased. This limited effect is in line with the present finding showing only partial consequences of MePD GABA_B_R antagonism together with kisspeptin optic stimulation.

Although the present study has shown a clear correlation between glutamatergic and GABAergic MePD activity and the GnRH pulse generator, it would be unwise to disregard the probability that there are other neurotransmitters at play, such as noradrenaline, in the amygdaloid modulation of gonadotropin pulsatility. Indeed, GABA release from interneurones has been shown to be enhanced by adrenergic receptor activation in the basolateral amygdala,^15^ and studies have indicated that kisspeptinergic activity in avoidance behaviour relies on several neurotransmitters, including through adrenergic transmission.^39^

These data have demonstrated, for the first time, the possible neuronal mechanisms by which increased kisspeptinergic activity within the amygdala increases GnRH pulse frequency, which could also provide the basis for the sexual development of puberty. It would be of interest to further investigate the roles of GABA and glutamate in this network, including in relation to perturbations of reproductive physiology associated with the amygdala such as stress and abnormal food intake.

## AUTHOR CONTRIBUTIONS

**Geffen Lass:** Conceptualization; data curation; formal analysis; investigation; methodology; writing – original draft; writing – review and editing. **Xiao Feng Li:** Conceptualization; data curation; formal analysis; investigation; methodology; writing – original draft; writing – review and editing. **Margaritis Voliotis:** Conceptualization; data curation; formal analysis; funding acquisition; investigation; methodology; writing – original draft; writing – review and editing. **Ellen Wall:** Data curation; investigation; methodology; writing – review and editing. **Ross de Burgh:** Investigation; methodology. **Deyana Ivanova:** Investigation; methodology; writing – review and editing. **Caitlin McIntyre:** Data curation; methodology. **Xian‐Hua Lin:** Data curation; investigation; methodology. **William Colledge:** Conceptualization; methodology; writing – review and editing. **Stafford Louis Lightman:** Conceptualization; writing – review and editing. **Krasimira Tsaneva:** Conceptualization; data curation; funding acquisition; methodology; writing – review and editing. **Kevin Thomas O'Byrne:** Conceptualization; funding acquisition; investigation; methodology; writing – original draft; writing – review and editing.

## CONFLICTS OF INTEREST

The authors declare that they have no conflicts of interest.

5

### PEER REVIEW

The peer review history for this article is available at https://publons.com/publon/10.1111/jne.13207.

## Data Availability

All data contained within the manuscript have been deposited in the King's Research Data Management System and are freely available to public access (www.kcl.ac.uk/library/researchsupport/research-data-management/preserve/deposit-your-data-with-kings3).
